# Role of Presynaptic Glutamate Receptors in Pain Transmission at the Spinal Cord Level

**DOI:** 10.2174/1570159X11311050002

**Published:** 2013-09

**Authors:** Rita Bardoni

**Affiliations:** Department of Biomedical, metabolic and neural sciences, University of Modena and Reggio Emilia, Italy

**Keywords:** Spinal cord, pain, glutamate, presynaptic modulation, primary afferent fiber, primary afferent depolarization.

## Abstract

Nociceptive primary afferents release glutamate, activating postsynaptic glutamate receptors on spinal cord dorsal horn neurons. Glutamate receptors, both ionotropic and metabotropic, are also expressed on presynaptic terminals, where they regulate neurotransmitter release. During the last two decades, a wide number of studies have characterized the properties of presynaptic glutamatergic receptors, particularly those expressed on primary afferent fibers. This review describes the subunit composition, distribution and function of presynaptic glutamate ionotropic (AMPA, NMDA, kainate) and metabotropic receptors expressed in rodent spinal cord dorsal horn. The role of presynaptic receptors in modulating nociceptive information in experimental models of acute and chronic pain will be also discussed.

## INTRODUCTION

Glutamate receptors(GluRs) are widely expressed in the membrane of spinal neurons postsynaptic to nociceptive afferents. In addition, subunits of both ionotropic and metabotropic GluRs are translated by dorsal root ganglion neurons (DRGs) and transported both peripherally and centrally. Peripheral transport of GluRs is suggested by immunoreactivity for NMDA, kainate, or AMPA receptors detected on nerve fibers and endings in the rodent and human skin [1-3]. Peripheral GluRs can be activated by application of agonists and function as sensors of glutamate released by peripheral terminals, contributing to sensitization after injury [4-6].

Central terminals of primary afferent fibers (PAFs) express both ionotropic and metabotropic GluRs. Their activation modulates the release of glutamate and peptides in dorsal horn (DH). Expression of presynaptic GluRs has been observed also on interneurons, particularly on GABAergic/glycinergic terminals. In the spinal cord DH, the flow of information from peripheral sensory receptors to the central nervous system is under constant regulation of presynaptic inhibition, *via *a mechanism known as primary afferent depolarization [7]. Although primary afferent depolarization is mainly mediated by GABA_A_ receptors, more recent studies have suggested the contribution of ionotropic GluRs [8]. The presence of functional ionotropic GluRs on PAF terminals supports the hypothesis that these receptors can actively control and modulate the transmission of nociceptive information at the first synapse. 

 In this review I will summarize the current knowledge about ionotropic and metabotropic GluRs expressed on axon boutons (“presynaptic GluRs”) in spinal cord DH, focusing in particular on the receptors located on PAF central axon terminals.

## AMPA RECEPTORS

AMPA receptors(AMPARs) are tetrameric assemblies of different combinations of four subunits, designated GluA1–GluA4(alternatively known as GluR1–GluR4 and GluR-A to GluR-D; reviewed in [9]). The Ca^2+^-permeability of AMPARs is prevented by the presence of the GluA2 subunit within the receptor. AMPARs have been detected on DRGs using immunohistochemistry and *in situ* hybridization [10, 11]. All four AMPAR subunits may be translated by DRG neurons. GluA1 is present on both unmyelinated and myelinated DRGs [10, 12], GluA2/3 seems to be predominant on myelinated neurons [13, 14], GluA4 is preferentially localized on unmyelinated fibers [13]. 

AMPAR subunits are transported to the central terminals of DRGs and are present in different DH laminae. As shown by electron microscopy studies performed on rat spinal cord sections, GluA4 is predominantly expressed in laminae I-III, often co-localized with the non-peptidergic fiber marker IB4, while GluA2/3 has been preferentially detected in laminae III-IV, on myelinated fibers [13]. Interestingly, AMPAR subunits have been identified also on presynaptic terminals of DH GABAergic interneurons [15]. 

Functional DRG AMPARs seem to be expressed mainly near the PAF terminals. Acutely dissociated DRGs do not show physiological responses following application of AMPAR agonists [16], while in embryonic co-cultures (obtained from E16 rats and recorded after 2-4 weeks, when DRGs extensively form synapses with DH neurons) 50% of DRGs exhibit responses to AMPA [12]. 

AMPARs on PAF central terminals mediate primary afferent depolarization and modulate glutamate release. In the hemisected rat spinal cord preparation, application of AMPA elicits the depolarization of the dorsal roots, blocked by AMPAR antagonists. The experiment has been performed in presence of tetrodotoxin and low extracellular Ca^2+^, to block synaptic transmission in the spinal cord [12]. In co-cultures of DRG and DH neurons, activation of AMPARs depresses excitatory postsynaptic currents (EPSCs) recorded from DH neurons and evoked by DRG stimulation [17]. We have shown that also EPSCs evoked by dorsal root stimulation in rat spinal cord slices, and recorded from lamina II neurons, are inhibited by the application of AMPAR agonists [12]. The interpretation of the experiments performed on slices, involving bath application of agonists, is not straightforward, due to the possibility of an indirect effect. Nevertheless, the increase of synaptic variability and the appearance of failures observed in this study suggest the presence of a presynaptic modulation [12, 18]. In transgenic mice carrying the deletion of the GluA1 subunit specifically on nociceptive DRGs, presynaptic inhibition by AMPARs is significantly inhibited. The deletion of GluA2 subunit does not have any significant effect [18], suggesting that presynaptic AMPARs on nociceptive PAFs are mainly calcium-permeable. AMPARs expressed on presynaptic terminals of DH inhibitory interneurons are also functional and able to modulate GABA and glycine release [19]. These receptors modulate in an opposite way the spontaneous and evoked release, causing the increase of frequency of miniature IPSCs (inhibitory postsynaptic currents) and the inhibition of evoked IPSCs.

The mechanism by which presynaptic AMPARs modulate glutamate, GABA and glycine release in DH has not been fully investigated. AMPARs might cause the decrease of evoked neurotransmitter release mainly by impairing the propagation of action potentials along the axon. Opening of AMPARs may shunt the action potential propagation by lowering the input resistance of the terminal, which decreases the magnitude of depolarization during the action potential and slows action potential propagation. Furthermore, AMPAR-mediated depolarization might cause the inactivation of voltage-dependent sodium channels, making them less available for action potential generation. A similar mechanism has been proposed for presynaptic GABA_A_ receptors, mediating primary afferent depolarization [20]. The facilitatory effect of presynaptic AMPARs on spontaneous release of GABA and glycine could be due to an increased opening of voltage-dependent Ca^2+^ channels, due to the terminal depolarization, and/or to Ca^2+^ entry directly through Ca^2+^-permeable AMPARs. 

While the effect of AMPARs expressed on peripheral DRG terminals on nociceptive behavior has been investigated, the role of receptors on central terminals has not been clearly established. Activation of AMPA and kainate receptors in glabrous skin of the rat hindpaw results in mechanical allodynia and mechanical hyperalgesia [21], while peripheral application of antagonists, specific for Ca^2+^-permeable AMPARs, alleviates inflammatory pain [18]. Specific deletion of GluA1 subunit in nociceptors decreases sensitization in inflammatory pain models (injection of Complete Freund Adjuvant into the hindpaw or knee arthritis), suggesting that both peripheral and central AMPARs could exert a pro-nociceptive action [18].

## KAINATE RECEPTORS

Kainate receptors (KARs) consist of tetrameric combinations of five subunits, forming two groups according to their low (GluK1 or GluR5, GluK2 or GluR6, GluK3 or GluR7), or high (GluK4 or KA1 and GluK5 or KA2) binding affinity. GluK1 is the predominant kainate receptor subunit expressed by DRG neurons [22] and its mRNA has been shown to be mostly localized in small-diameter DRGs [10]. Smaller amounts of other subunits have also been detected, including GluK2, GluK3, GluK4, and GluK5 [22]. Of the DRG neurons that test positive for kainate responses, 92% are positive for IB4, showing that they are non-peptidergic [23]. So, differently from AMPARs, KARs are mostly expressed by nociceptors (C fibers). Due to the developmentally-regulated RNA editing of the GluK1 subunit, DRG neurons from late embryonic and newborn rats are predominantly Ca^2+^- permeable but then become fully Ca^2+^- impermeable later in the first postnatal week. Calcium-permeable KARs on C-fiber growth cones could function as a signal for growing C fibers to stop in superficial DH and form synapses [24].

The prevalence of the GluK1 subunit in DRGs is also confirmed by several electrophysiological studies. Application of kainate or ATPA (a GluK1-selective agonist) on freshly dissociated or cultured DRGs elicits a rapidly desensitizing current in the majority of small diameter neurons [16]. In mice lacking the GluK1 subunit, the DRG response to kainate is strongly reduced [25]. In contrast, a significant reduction of KAR-mediated current density in DH neurons is observed only after deletion of the GluK2 subunit, whereas GluK1deletion causes no change [25].

In rat spinal cord DH, staining for GluK1/K2/K3 (mostly due to GluK1) has been observed on the terminals of a large number of PAFs, both unmyelinated and myelinated. Nociceptive afferent terminals belong mostly to non-peptidergic fibers [26, 27]. High affinity subunits (GluK4 and GluK5) have also been detected on nociceptive non-peptidergic fibers [27]. Similarly to AMPARs, presynaptic terminals of GABAergic interneurons express kainate receptor subunits, especially in lamina I-III of the DH [28].

Activation of KARs expressed on the central PAF terminals generates dorsal root depolarization and depresses compound action potentials, selectively on C fibers [12, 29, 30]. Presynaptic KARs on PAF terminals modulate glutamate release in DH, similarly to AMPARs. A decrease of evoked glutamate release has been demonstrated in co-cultures of DRG and DH neurons and in spinal cord slices [17, 31]. Although a metabotropic mechanism has been suggested for KARs expressed on DRGs [32], the modulatory effect on transmitter release is likely due to an ionotropic mechanism, similar to AMPA receptors. The effect is mediated by GluK1-containing receptors, since it is observed after application of ATPA and is reduced in mice lacking of GluK1 [25]. KARs expressed on GABAergic terminals, activated by kainate or glutamate (but not ATPA), increase spontaneous, action-potential independent, GABA and glycine release and inhibit evoked release. The latter effect could be due to the activation of GABA_B_ autoreceptors [33]. 

Behavioral studies have confirmed a modulatory role of GluK1-expressing KARs on pain transmission, suggesting the involvement of DRG KARs in pain sensitization. Intraperitoneal administration of a selective GluK1 antagonist significantly attenuates the late phase of formalin-induced paw-licking behavior [34]. Antiallodynic and antihyperalgesic effects of selective GLUK1 antagonists have also been described in the capsaicin and carrageenan models in rat [35]. Accordingly, nociceptive responses to capsaicin or formalin are significantly reduced in mice lacking GLUK1, but not GluK2, subunits [36].

## NMDA RECEPTORS

NMDA receptors(NMDARs) exist as tetramers, typically composed of two GluN1 (or NR1) and two GluN2 subunits [9]. The GluN2 subunits consist of four different types (GluN2A–D, or NR2A-D): the expression of different GluN2 subunits regulates several receptor properties, such as kinetics and magnesium sensitivity.

Both large and small DRGs express GluN1 mRNA [37, 10]. The subunit is present in both peptidergic and non-peptidergic nociceptive DRGs [14]. Immunohistochemistry and molecular biology studies have shown that GluN2B and D predominate on GluN2A and C in DRG neurons. While the most common NMDA receptor in A fibers seems to be the diheteromer GluN1/GluN2B, C fibers could also express an additional receptor containing GluN1, GluN2D, and possibly GluN2C subunits [38, 39]. 

Presynaptic NMDARs on PAF terminals of the spinal cord are translated in the DRG cells and transported centrally along the dorsal root [40]. The majority of immunostained terminals for GluN1 detected in laminae I-IV have morphological characteristics of PAF endings and most of them are myelinated fibers [41]. Analogously to the other ionotropic GluRs, NMDARs are also present on GABAergic terminals in laminae I-III [28]. 

Application of glutamate or NMDA, in absence of extracellular magnesium, evokes an inward current and the increase of intracellular calcium in most DRGs [42, 43]. The GluN2B antagonist ifenprodil blocks 94% of the NMDA induced current in adult rat DRGs, confirming that functional NMDARs are composed predominantly of GluN2B subunit. Interestingly, the average density of NMDA current is 2.8-fold larger in DRG neurons from female rats compared with male rats. Addition of 17-β-estradiol potentiates NMDA currents, particularly in female neurons [44].

In addition to AMPA and KARs, also NMDARs contribute to primary afferent depolarization. Application of NMDA on the rat hemisected spinal cord generates dorsal root depolarization, blocked by the NMDA antagonist D-APV [45]. The primary afferent depolarization is observed in presence of 1 mM extracellular magnesium, suggesting that NMDARs expressed on PAF terminals are not completely blocked by magnesium ions at the resting membrane potential.

 Presynaptic NMDARs on central PAF terminals are involved in modulation of neurotransmitter release. Earlier studies, performed on rat spinal cord slices, showed that their activation induces the release of substance P from nociceptive PAFs and the internalization of NK1 receptors in DH lamina I [46, 47]. Stimulation of dorsal root at high frequency (100 Hz) also causes the release of substance P and NK1 internalization, blocked by NMDA receptor antagonists. PAF depolarization produced by high frequency stimulation, together with a massive release of glutamate, activates NMDA autoreceptors [48]. Blockade of voltage-dependent sodium or calcium channels does not prevent the effect, indicating that Ca^2+^ entering NMDARs is sufficient to induce substance P release from PAF terminals [49]. Results from experiments *in vivo* are controversial, failing to show, in some cases, an effect of NMDA administration on substance P release and NK1 receptor internalization [50, 51]. The reasons for these discrepancies between *in vivo *and *in vitro* studies are still not clear and need to be investigated by future experiments.

 In addition to substance P, presynaptic NMDARs might contribute to the release of the neurotrophin BDNF from nociceptive PAFs [52]. Short bursts of high-frequency stimulation of the dorsal root evoke release of BDNF, along with substance P and glutamate, in rat spinal cord slices. The effect is blocked by D-APV.

 We have shown that presynaptic NMDARs could modulate glutamate release from PAFs in rat spinal cord slices [45]. Application of NMDA causes the depression of evoked EPSCs (accompanied by an increase of synaptic latency) in lamina II neurons from postnatal rats, suggesting that NMDARs interfere with action potential propagation along PAFs. Zeng *et al*. [53] have reported that NMDA application reduces the frequency of miniature EPSCs in spinal cord slices from neonatal rats. Interestingly, presynaptic NMDARs exert an opposite action in opioid-tolerant animals, causing the increase of miniature, spontaneous and evoked EPSCs. An increased expression of NMDARs in substance P-containing PAF terminals in DH has also been observed [53]. The facilitating effects of presynaptic NMDARs after opiate exposure are blocked by PKC inhibitors, suggesting that increased PKC activity could potentiate presynaptic NMDA receptor function by reducing magnesium sensitivity and promoting receptor trafficking to the plasma membrane [54]. Similar results have been recently reported by Yan *et al*. [55], showing a facilitatory action of NMDARs (both exogenously or endogenously activated) on glutamate release from PAFs in adult neuropathic rats. The increased expression of NMDARs located on PAFs and expressing the GluN2B subunit seems to play a critical role in this effect.

The role of DRG NMDARs in different animal models of pain has been investigated. Opening of peripheral receptors by intraplantar NMDA injection causes the increase of c-fos expression in superficial DH [56], indicating that they can mediate the activation of PAFs. Local cutaneous administration of NMDA receptor antagonists significantly inhibits phase 2 but not phase 1 response to subcutaneous formalin [57, 58]. Accordingly, mice lacking the GluN1 subunit in peripherin positive cells (whose DRGs exhibit a decrease of 75% of GluN1 expression with no change in CNS) show a significant decrease of phase 2 formalin-induced nociceptive behavior, while acute pain is unaffected [59]. NMDARs expressed on DRGs could contribute to generate pain sensitization at the spinal cord level by producing a continuous activation of PAFs and/or by increasing the release of substance P and other pain mediators from PAF central terminals.

Chronic exposure to opioids causes analgesic tolerance and hyperalgesia. As already mentioned, experiments performed *in vitro* have shown that presynaptic NMDARs expressed on PAFs are involved in these effects, by potentiating glutamatergic transmission in DH [53]. Intrathecal injection of D-APV prevents the development of analgesic tolerance and hyperalgesia induced by chronic morphine in adult rats [54]. This effect, that could also involve a direct action of D-APV on DH neurons, is consistent with the proposed role of NMDARs expressed on PAF terminals.

## METABOTROPIC GLURS

Metabotropic GluRs (mGluRs) are represented by eight receptors divided into three groups: group 1 with mGluR1 and mGluR5, group 2 including mGluR2 and mGluR3, and finally group 3 with mGluR4, mGluR6, mGluR7, and mGluR8. In DRG neurons group 3 has the highest expression, followed by group 2 and group 1. Staining for all mGluRs (with the exception of mGluR6) has been detected on DRGs, with a prevalence for mGluR8 (75% of DRGs) and mGluR2/3 (likely only mGlu2, in about 50% DRGs) [60]. mGluR8 is expressed by small, medium, and large diameter DRGs. In contrast, mGluR2/3 (and mGluR1α) is mainly expressed by small and medium neurons. 

Labelling for different mGluRs has been observed also on central terminals of PAFs, although the results are sometimes controversial. Staining for mGluR2/3 has been detected in DH, preferentially in lamina II. Dorsal root rhizotomy eliminates part of labeling suggesting that it is partially due to mGluRs on PAFs [61]. However, other studies have reported that Glu2/3 staining in DH is prevalently due to mGluRs expressed on interneurons, particularly on GABAergic terminals [62, 63]. Immuno-reactivity for mGluR4 and mGluR7 has been found in the superficial DH, especially on PAF terminals [64-66].

Group 2 and 3 mGluRs are typically coupled to inhibitory Gi/o proteins, thereby inhibiting cAMP formation and voltage-gated Ca^2+^ channels and decreasing neuro-transmitter release in CNS. In rat spinal cord slices, agonists of these mGluRs produce a decrease of monosynaptic and polysynaptic EPSPs recorded from DH neurons [67]. Activation of mGluRs causes a significant change of paired pulse ratio (the ratio between two consecutive synaptic responses), indicating a presynaptic effect. Zhang *et al*. [68] have recently shown that application of group 3 mGluR agonist L-AP4 on spinal cord slices has a greater inhibitory effect on the amplitude of monosynaptic and polysynaptic eEPSCs in nerve-injured rats than in control animals. L-AP4 depresses also GABA and glycine release, but no differences have been observed between the two animal groups. The concurrent effect of III mGluR agonists on both excitatory and inhibitory synaptic transmission in spinal cord DH may explain the lack of the net effect of L-AP4 on nociception in normal animals [69]. However, intrathecal administration of L-AP4 reduces nociceptive responses induced by formalin injection in rats [70] and attenuates allodynia in rats subjected to spinal nerve ligation [69]. Up-regulation of group 3 mGLURs (similarly to what observed for group 2 mGluRs, [71]), occurring in conditions of pain sensitization, could explain these results. 

## CONCLUSIONS AND PERSPECTIVES

**Fig. (1):**
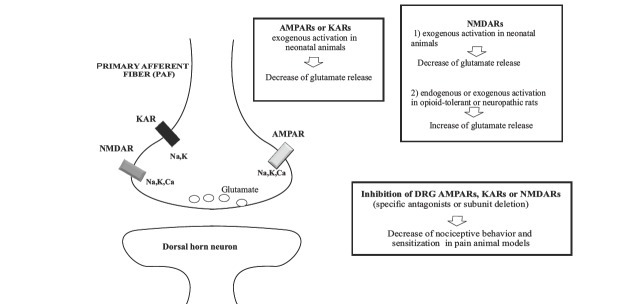
Ionotropic GluRs located on central terminals of PAFs. *Left*: Schematic diagram representing the different GluR types and their ionic permeability. *Right*: ) Effects of GluR activation or inhibition on glutamate release from PAFs (top diagrams, *in vitro* studies) and pain sensitivity (bottom diagram, behavioral experiments).

Despite the large amount of studies describing the composition and functional properties of presynaptic GluRs in spinal cord (summarized in Fig. **1**), there are several aspects that still need to be clarified. 

The first issue is related to the physiological action of ionotropic GLURs expressed on PAF and interneuron terminals in spinal cord DH. Most studies have been performed by exogenously applying specific receptor agonists, at concentrations that could be different from those physiologically reached during synaptic activity. It has been shown that presynaptic ionotropic receptors can exert a biphasic modulation, depending on the ligand concentration. At low agonist concentrations a small depolarization is produced, sufficient to facilitate action potential generation and facilitation of evoked release. On the contrary, high concentrations induce larger depolarizations, responsible for action potential inhibition and depression of release [72]. In rat spinal cord slices, the increase of endogenous glutamate, obtained by blocking a glutamate transporter, produces the rise of miniature EPSC frequency [73]. This suggests that presynaptic GLURs receptors might enhance glutamate release when endogenously activated, while they inhibit release when high concentrations of agonists are applied. The use of transgenic mice with deletion of GluR subunits in specific population of PAFs will be very helpful to clarify the role of these receptors during endogenous activation in physiological and pathological conditions. 

Another important aspect is the identification of the source of glutamate activating presynaptic GluRs in DH. Spillover of glutamate released by nearby glutamatergic synapses (belonging to PAFs or excitatory interneurons) could activate presynaptic receptors and induce modulation of transmitter release. This mechanism might be particularly important during intense activation of glutamatergic inputs and/or when glutamate uptake is altered, conditions that often occur during central pain sensitization. Differently from GABA and glycine, glutamatergic synaptic contacts on PAFs do not seem to be frequent in rodent spinal cord [74], suggesting that the release of glutamate at axo-axonic synapses may not be relevant for presynaptic GluR activation. 

An additional source of glutamate could be represented by glial cells, particularly astrocytes. In DH and other CNS areas, elevations of intracellular Ca^2+^ in astrocytes evokes glutamate release, able to activate glutamatergic receptors on nearby neurons [75, 76]. Further experiments are necessary to understand which pain states are able to activate astrocytic glutamate release and what is the role of presynaptic GluRs in this mechanism. 

Finally, the development of analgesics drugs targeting spinal presynaptic receptors has been often prevented by the widespread expression of these receptors in the CNS. The investigation of the subunit composition of presynaptic receptors expressed on identified DH synapses will be important to identify new and more specific therapeutic treatments.
